# Outage Performance Analysis of NOMA in Wireless Powered Cognitive Radio Networks with AF and DF Relaying Techniques

**DOI:** 10.3390/e23111463

**Published:** 2021-11-05

**Authors:** Hui Wang, Jian Dong, Kun Tang, Heyuan Shi

**Affiliations:** 1 School of Computer Science and Engineering, Central South University, No. 932 Lushannan Road, Changsha 410083, China; wanghui0517@csu.edu.cn (H.W.); dongjian@csu.edu.cn (J.D.); 2 Guangdong Provincial Key Laboratory of Millimeter-Wave and Terahertz, School of Electronic and Information Engineering, South China University of Technology, No. 381 Wushan Road, Guangzhou 510640, China; tangkun@scut.edu.cn; 3 College of Intelligent Manufacturing, Hunan University of Science and Engineering, Yongzhou 425000, China; 4 School of Software, Tsinghua University, Beijing 100084, China

**Keywords:** relay-assisted NOMA, cognitive radio network, energy harvesting, amplified-and-forward relaying, decoded-and-forward relaying

## Abstract

Improving spectral efficiency under a certain energy limitation is an important design metric for future wireless communications as a response to the growing transmission demand of wireless devices. In order to improve spectral efficiency for communication systems without increasing energy consumption, this paper considers a non-orthogonal multiple access (NOMA)–based cognitive radio network, with the assistance of a wireless-powered relay station (RS), and then analyzes the system outage performance under amplified-and-forward (AF) and decoded-and-forward (DF) cooperative transmission modes. Specifically, the base station (BS) has the opportunity to cooperate by transmitting information through the RS, depending on whether the RS can harvest sufficient RF energy for cooperative transmission. That is to say, when the energy stored by the RS is sufficient for cooperative transmission, the RS will assist the BS to forward information; otherwise, the BS will send information through direct links, while the RS converts the radio frequency (RF) signals sent by the BS into energy for future transmission. Moreover, the transmission power required by the RS for cooperative transmission is usually relatively large, while the amount of harvested energy by the RS in a transmission slot is usually low, so it takes several consecutive time slots to accumulate enough transmission energy. To this end, we utilize a discrete-time Markov chain to describe the processes of charging and discharging of the RS. Subsequently, we derive the closed-form outage probabilities of both the primary and secondary systems for the considered system in AF and DF modes through mathematical analysis, and verify the accuracy of the analyses through Monte Carlo simulation. The simulation results show that the two proposed cooperative transmission schemes with AF and DF relaying techniques outperform both direct transmission and other similar schemes in both the primary and secondary system, while the DF scheme can provide better performance than the AF scheme within the range of setting values.

## 1. Introduction

With the development of communication technology, service demand has increased, which requires communication systems to achieve low energy consumption and high spectrum efficiency to support various services. Researchers have paid more attention to non-orthogonal multiple access (NOMA) in the last few years [1], which is a valuable multiple access technique for fifth generation (5G) wireless networks. With NOMA, user equipments (UE) superpose the messages in the same time/frequency/code/power domain and impose inter-user interference simultaneously at the transmitter side, while the receivers side employs the successive interference cancellation (SIC) technique [2,3]. All these attractive characteristics of NOMA can provide higher spectrum efficiency for the overall system and better fairness for users [4]. Furthermore, cooperation communication between nodes can expand network coverage and further improve the diversity and performance of the system. Therefore, the cooperative NOMA (C-NOMA) [5,6] scheme can further enhance the performance of NOMA, where one NOMA user acts as a relay to help the other one for reliable transmission.

Another way to improve spectrum efficiency is through a cognitive radio network (CRN), also known as dynamic spectrum access, which is aware of the surroundings and has the capability to adapt its transmission characteristics. In order to improve the utilization of spectrum resources, CRNs operate on the spectrum cooperation between primary users (PUs) and secondary users (SUs), so SUs and PUs can utilize the same frequency band. SUs can opportunistically use the spectrum of PUs for data transmission, as long as SUs meet certain constraints to ensure the communication quality of PUs [7,8].

Radio frequency energy harvesting (RF-EH) significantly prolongs the network life and improves the efficiency of energy utilization, which uses receiving antennas to harvest RF signals sent by other sources and then converts the collected signals into electronic power [9]. Therefore, the combination of CRN, C-NOMA, and energy harvesting techniques obviously provide the higher spectrum efficiency and better outage performance.

### 1.1. Related Work

According to the analysis above, C-NOMA overcomes the problem of poor spectral efficiency, which has been investigated widely. In [10], the authors proposed a simple scheme to select a near user (NU) and a far user (FU) and switch between orthogonal multiple access (OMA) and NOMA so as to significantly improve the FU’s performance without affecting the NU’s performance. Analyses show that if the selected near user adopts the SIC technique and forwards the FU’s signals when NOMA is used, FUs and NUs can achieve diversity gains of N+F and N, respectively, when an optimal combination is performed. A cooperative NOMA system with buffer-aided relaying was considered in [11], where the authors proposed an adaptively optimal selection scheme for the transmission mode. Numerical results validated that the proposed scheme can effectively improve the system throughput. In [12], the authors investigated the performance of a NOMA-assisted cooperative relaying system, where two transmitters used a shared AF relaying scheme to communicate with their corresponding receivers. The simulation results demonstrated that the proposed scheme significantly outperformed the conventional OMA techniques. The outage performance of a downlink C-NOMA scenario by adopting an AF relay was analyzed in [13], which can achieve the same diversity order and superior coding gain, compared to the conventional OMA scheme. The authors in [14] investigated the outage performance of spectrum sharing–based cooperative NOMA networks with DF relaying mode over Nakagami-m channels for different impact of fading parameters, where two source were paired to perform NOMA transmission to two paired destination nodes. A novel scheme for CRNs based on NOMA was proposed in [15], which applied the DF relaying scheme to transmit a signal for a SU and considered both spectrum sensing and transmission phases jointly. For characterizing the system performance, the authors derived the outage probability and ergodic rate of the system and demonstrated the effectiveness of the proposed scheme. The authors of paper [16] proposed a resource collaborative protocol for NOMA-based cooperative CRNs, which adopted maximum and minimum relay selections to improve both quality of service (QoS) and priority. The qualitative numerical results showed that the proposed optimal resource allocation strategy can significantly enhance the sum transmission rate and transmission performance of the secondary system when compared to a traditional CRN-NOMA system. The authors of [17] developed a DF-based cooperative underlay CR-NOMA network which considered the case of imperfect channel state information on the primary network for all secondary transmit nodes. The simulation results also demonstrated its superiority compared with cooperative OMA scheme. A two-hop DF cooperative underlay CR-NOMA network was described in [18], where it used an independent but necessarily identical distributed Nakagami-m fading channels model and imposed an interference temperature constraint at the relay of the secondary system to reduce the occurrence probability of serious interference at the PU. In recent years, the combination of CR-NOMA and the wireless power technique has attracted widespread attention. The authors in [19] illustrated a full duplex DF cooperative NOMA network, where the relay can recycle its energy from the self-loop interference channel based on the SWIPT technique. The results illustrated that energy harvesting in cooperative NOMA can improve the outage performance significantly. A SWIPT-based CR-NOMA systems was investigated in [20] in which the authors formulated the optimization problem for maximizing the throughput of the overall system, and then proposed a corresponding algorithm based on the dichotomy method to jointly realize the optimal allocation of the time slot and transmission power. In [21], the authors considered a SWIPT-based CRN with the fixed power allocation strategy of the NOMA scheme (SWIPT-F-NOMA), where the cognitive relay first harvested RF energy from the secondary transmitter and then cooperatively relayed the signal to the destination. The authors in [22] proposed a transmission scheme in a multi-user multiple input multiple output (MIMO) configuration, which adopted the code reuse technology.

### 1.2. Contribution

In the above literatures, there are few studies on the use of DF and AF relay schemes in NOMA-based CR networks with the energy harvesting technique. Moreover, the outage performance of NOMA-based CR networks with the energy harvesting technique, especially for NOMA-based CR networks with the discrete-time energy harvesting technique, has not been well studied. Generally, the energy harvesting only lasts for one transmission time slot, and the amount of harvested energy is small in one transmission time slot, which usually cannot satisfy the needs for a cooperative transmission. Thus, we propose two cooperative transmission schemes for the proposed NOMA-based CR networks, where the RS obtains sufficient energy in consecutive time slots and then forwards the signal by utilizing DF or AF relaying techniques. The main contributions of this work are summarized as follows:

- Based on AF and DF cooperative transmission modes, we propose two efficiently cooperative transmission schemes for a NOMA-based cooperative CRN, where relay node RS can harvest RF energy from the primary transmitter and perform cooperative transmission for the primary system when it accumulates sufficient energy; otherwise, the RS continues to harvest energy for future use. In our proposed schemes, the RS opportunistically participates in the cooperative transmission and the link diversity gain is increased under the condition that the transmitter power of the sender is unchanged; therefore, the spectral efficiency and energy efficiency of the system can be effectively improved.
- The battery size of the RS is limited, and the battery of RS usually needs to go through several continuous time slots to accumulate enough transmission energy. We model the dynamic behavior of charging and discharging of RS’s battery as a discrete Markov chain (MC) with a finite state, which can provide the exact probability of the RS participating in cooperative transmission within a transmission slot, according to the analytical expressions of state transition probabilities under AF and DF relaying schemes.
- We derive the analytical expressions of outage probabilities for both the primary and secondary systems under AF and DF relaying protocols, respectively. The authenticity and validation of derived outage probabilities are examined with the aid of Monte Carlo simulations. According to the numerical results of the derived outage expressions, it provides a practical guideline that the proposed spectrum sharing schemes can be significantly impacted by various system parameters, such as system power allocation and transmission power, and confirms the superiority of the proposed schemes over direct transmission and the scheme of SWIPT-F-NOMA.

The rest of this paper is organized as follows. In Section 2, the system model and two transmission schemes based on the NOMA-assisted CR network are introduced. Section 3 exhibits the transmission protocol with the AF relaying technique, where we first analyze the process of discrete-time energy accumulation and then discuss the outage probability with the AF relaying scheme. Correspondingly, the performance with DF relaying scheme is illustrated in Section 4. Section 5 contains the numerical simulations. Finally, the conclusion of this paper is drawn in Section 6.

## 2. System Model and Transmission Protocols

**Figure 1:**
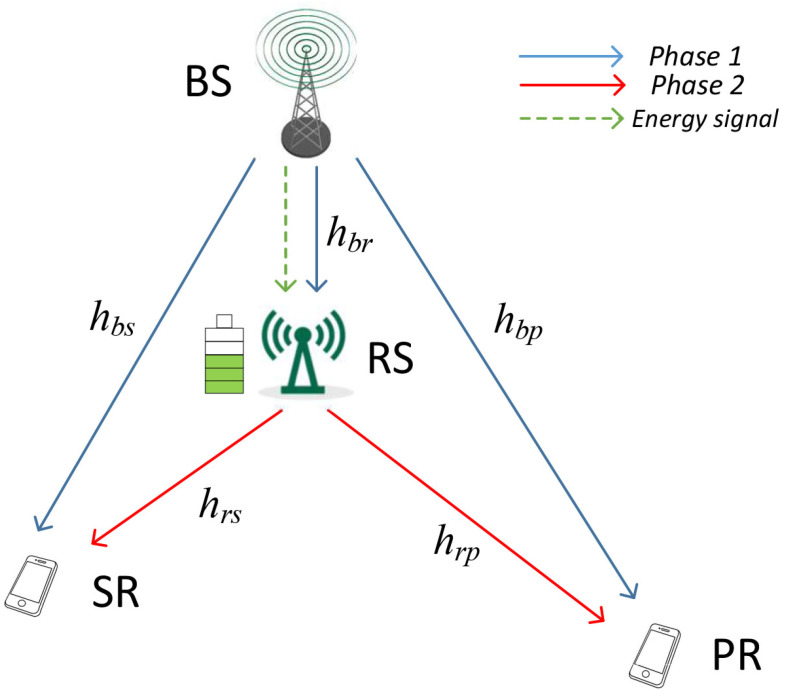
System model.

This paper considers a relay-assisted CR network based on NOMA as shown in Figure 1, in which BS conveys the message to both the PR and SR with/without the assistance of RS. For practical applications, we suppose that the RS has a battery with a limited capacity to store the energy harvested from BS’s signal. The RS can also opportunistically perform the spectrum sharing if the accumulated energy of RS’s battery is sufficient. Moreover, we assume that each node (BS, RS, SR, and PR) has a single antenna which works at half-duplex mode. It is assumed that channels are the quasi-static Rayleigh fading channels in the system. The channel coefficients between BS and the other nodes (PR, RS, and SR) are denoted as $h_{bp}$, $h_{br}$, and $h_{bs}$, while the channel coefficients between RS and the receiver nodes (PR and SR) are expressed as $h_{rp}$ and $h_{rs}$, respectively. Hence, let $|h_{i}{|}^{2}∼\mathrm{CN}\left(0,\lambda_{i}\right)\left(i=bp,br,bs,rp,rs\right)$ represents the power gain of channel.

In the above system model, at the start of each time block, the RS judges whether its stored energy is sufficient, and if so, it first broadcasts the request to send (RTS) frame and then enters the mode of cooperative transmission in the next slot. Otherwise, the RS switches to the energy harvesting mode, and the BS directly sends its information to both the PR and SR in the next block. In the cooperative transmission mode, the transmission block is divided into two phases with equal time. During the first phase, BS transmits a composite signal $x\left(t_{1}\right)=a_{p}x_{p}+a_{s}x_{s}$ to RS, PR, and SR, where $x_{p}$ and $x_{s}$ denote signals desired by PR and SR, respectively, and $a_{s}$ and $a_{p}$ denote power distribution coefficients correspondingly. NOMA technology ensures more transmit power for PR’s signal and less power for SR’s signal with $a_{p}^{2}+a_{s}^{2}=1\left(a_{s}<a_{p}\right)$ [23]. Hence, the received signals at RS, SR and PR can be given by the following:

(1) $$\begin{array}{c} y_{p}\left(t_{1}\right)=\sqrt{P_{BS}}\left(h_{bp}\left(a_{p}x_{p}+a_{s}x_{s}\right)\right)+n_{bp},\\ y_{s}\left(t_{1}\right)=\sqrt{P_{BS}}\left(h_{bs}\left(a_{p}x_{p}+a_{s}x_{s}\right)\right)+n_{bs},\\ y_{r}\left(t_{1}\right)=\sqrt{P_{BS}}\left(h_{br}\left(a_{p}x_{p}+a_{s}x_{s}\right)\right)+n_{br}, \end{array}$$

respectively, where $n_{i}∼\mathrm{CN}\left(0,\delta^{2}\right)\left(i=bp,bs,br\right)$ interprets the received additive white Gaussian noise (AWGN), and $P_{BS}$ denotes BS’s transmission power.

Correspondingly, in the first phase, the signal to interference and noise ratio (SINR) at PR is expressed as follows:

(2) $$\gamma_{bp}=\frac{a_{p}^{2}P_{BS}{\left|h_{bp}\right|}^{2}}{a_{s}^{2}P_{BS}{\left|h_{bp}\right|}^{2}+\delta^{2}}.$$

Based on decoding scheme of NOMA, the SR first detects signal of PR and then utilizes SIC technique to eliminate it for obtaining its own signal. The SINRs for SR detecting the signal of PR and SR are given by the following:

(3) $$\gamma_{bs1}=\frac{a_{p}^{2}P_{BS}{\left|h_{bs}\right|}^{2}}{a_{s}^{2}P_{BS}{\left|h_{bs}\right|}^{2}+\delta^{2}},$$

(4) $$\gamma_{bs2}=\frac{a_{s}^{2}P_{BS}{\left|h_{bs}\right|}^{2}}{\delta^{2}},$$

respectively.

Similarly, the SINRs of RS detecting signals from PR and SR are successively derived as follows:

(5) $$\gamma_{br1}=\frac{a_{p}^{2}P_{BS}{\left|h_{br}\right|}^{2}}{a_{s}^{2}P_{BS}{\left|h_{br}\right|}^{2}+\delta^{2}},$$

(6) $$\gamma_{br2}=\frac{a_{s}^{2}P_{BS}{\left|h_{br}\right|}^{2}}{\delta^{2}}.$$

Considering that PR and SR are far from BS, the channels from BS to PR and SR show large-scale path fading. During the second transmission phase, RS acts as a relay by using the DF or AF relaying technique to convey the combined message cooperatively, which is received from BS in the first phase only if it has sufficient energy.

### 2.1. AF Relaying Scheme

**Figure 2:**
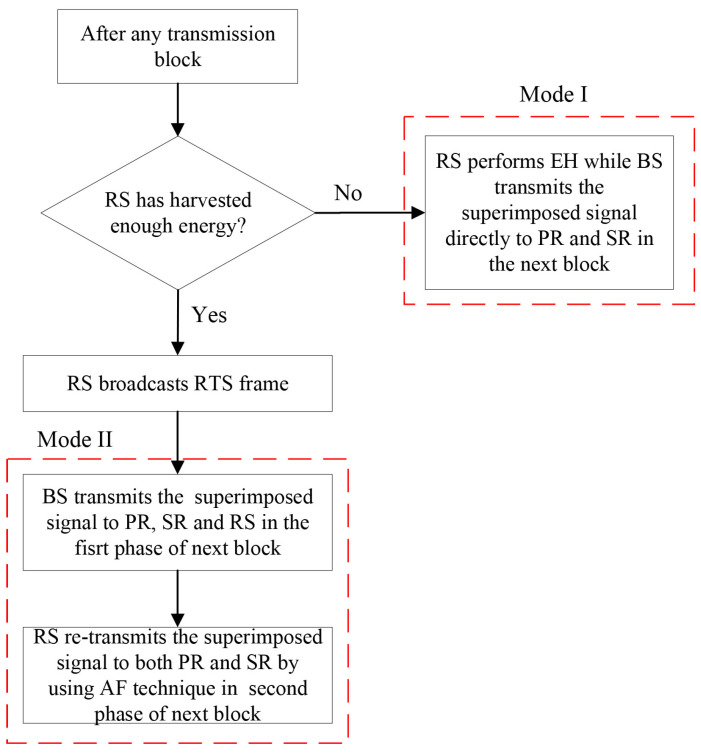
Flow chart of AF relaying scheme.

When the system adopts the AF relaying technique in the second phase, RS amplifies the received signal and forwards it to PR and SR after it receives BS’s signal. PR obtains its own message by treating SR’s message as interference, while SR subtracts the desired message by applying the SIC technique. Therefore, the operation process of AF relaying scheme is shown as Figure 2, where the network opportunistically performs one of two modes in one time block: (1) Mode I: when RS has not harvested enough energy, RS continues to perform energy harvesting while BS transmits the superimposed signal to both PR and SR directly in next time block; (2) Mode II: when RS has harvested enough energy, it broadcasts the RTS frame and then relays the superimposed signal from BS by adopting the AF relaying technique in the next time block.

In Mode I, the residual energy of RS’s battery is less than the energy threshold value $E_{T}$. BS directly conveys the signal to both PR and SR while RS harvests energy in the next transmission block. The amount of harvested energy at RS can be derived as the follows:

(7) $$E_{H}^{AF}=\eta TP_{BS}\left|h_{br}\right|{}^{2},$$

where 0≤η≤1 denotes the energy conversion efficiency, and T=1 represents the normalized transfer time in the following.

Mode II corresponds that the residual energy of RS’s battery exceeds or is equal to $E_{T}$. Then, RS sends RTS to all nodes and acts as AF relaying to re-transmit the combined signal $x(t_{2})$ to PR and SR in the second phase. The combined signal $x(t_{2})$ can be expressed as the following:

(8) $$x\left(t_{2}\right)=Gy_{r}\left(t_{1}\right)=G\sqrt{P_{BS}}\left(h_{br}\left(a_{p}x_{p}+a_{s}x_{s}\right)\right)+Gn_{br},$$

where we assume that the thermal noise generated by the signal conversion can be neglected. Therefore, the power amplification factor *G* is given by the following:

(9) $$G=\sqrt{\frac{1}{P_{BS}{\left|h_{br}\right|}^{2}+\delta^{2}}}.$$

Therefore, the signals received by PR and SR are written as follows:

(10) $$\begin{array}{cc} y_{p}^{AF}\left(t_{2}\right) & =\sqrt{P_{RS}}h_{pr}x\left(t_{2}\right)+n_{pr}\\ & =G\sqrt{P_{RS}}\sqrt{P_{BS}}h_{pr}h_{\mathrm{br}}\left(a_{p}x_{p}+a_{s}x_{s}\right)+Gn_{br}h_{pr}+n_{pr}, \end{array}$$

(11) $$\begin{array}{cc} y_{s}^{AF}\left(t_{2}\right) & =\sqrt{P_{RS}}h_{rs}x\left(t_{2}\right)+n_{rs}\\ & =G\sqrt{P_{RS}}\sqrt{P_{BS}}h_{rs}h_{\mathrm{br}}\left(a_{p}x_{p}+a_{s}x_{s}\right)+Gn_{br}h_{rs}+n_{rs}, \end{array}$$

respectively, where $P_{RS}$ represents transmission power of RS. According to the above formulas, the SINR at PR during the second phase is expressed as follows:

(12) $$\gamma_{rp}^{AF}=\frac{\alpha \beta a_{p}^{2}{\left|h_{rp}\right|}^{2}\left|h_{br}\right|{}^{2}}{\alpha \beta a_{s}^{2}{\left|h_{rp}\right|}^{2}\left|h_{br}\right|{}^{2}+\alpha \left(\left|h_{rp}\right|{}^{2}+\left|h_{br}\right|{}^{2}\right)+1},$$

where $\alpha =\frac{P_{BS}}{\delta^{2}},\beta =\frac{P_{RS}}{\delta^{2}}$. The SIC technique is applied at SR, which detects PR’s message signal and then cancels that message signal from its own observation. Thus, the SINRs at SR for detecting the signal of PR and SR are expressed as follows:

(13) $$\gamma_{rs1}^{AF}=\frac{\alpha \beta a_{p}^{2}{\left|h_{rs}\right|}^{2}\left|h_{br}\right|{}^{2}}{\alpha \beta a_{s}^{2}{\left|h_{rs}\right|}^{2}\left|h_{br}\right|{}^{2}+\alpha \left(\left|h_{rs}\right|{}^{2}+\left|h_{br}\right|{}^{2}\right)+1},$$

(14) $$\gamma_{rs2}^{AF}=\frac{\alpha \beta a_{s}^{2}{\left|h_{rs}\right|}^{2}\left|h_{br}\right|{}^{2}}{\alpha (\left|h_{rs}\right|{}^{2}+\left|h_{br}\right|{}^{2})+1},$$

respectively.

### 2.2. DF Relaying Scheme

**Figure 3:**
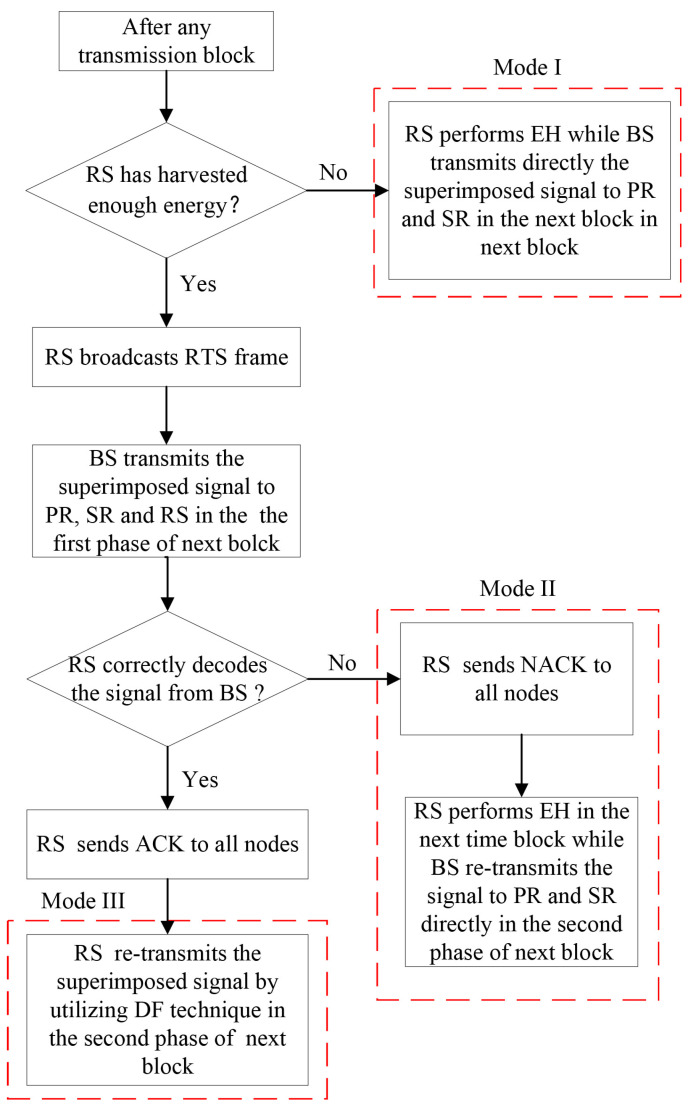
Flow chart of DF relaying scheme.

When the system adopts the DF relaying scheme during the second phase, the RS first decodes the received composited signal from the BS. If the RS decodes the message successfully, it re-encodes the composited signal and transmits the re-encoded signal to both the PR and SR, which extract their own information by employing the SIC technique. If unsuccessful, the BS re-transmits the signal. Therefore, there are three modes under the DF relaying scheme as shown in Figure 3. (1) Mode I: If RS has not harvested enough energy, it keeps harvesting energy while the BS directly conveys the composited signal to both PR and SR in next transmission block; (2) Mode II: If the RS has accumulated enough energy but the received signal is decoded incorrectly, the NACK frame is sent to all nodes from RS while the energy harvesting mode remains unchanged, and then BS directly re-transmits the composited signal to both PR and SR; (3) Mode III: If RS has not only accumulated sufficient energy but also correctly decoded the comprised signal from BS, it sends ACK to all nodes and then transmits the re-coded signal.

In Mode I, the amount of residual energy of RS’s battery is less than $E_{T}$ and then the RS continues to harvest energy. Thus, the amount of harvested energy can be written as follows:

(15) $$E_{H}^{DF,I}=\eta TP_{BS}\left|h_{br}\right|{}^{2}.$$

Mode II corresponds that the composited signal is incorrectly decoded at the RS, whose amount of remaining energy is greater than or equal to the threshold $E_{T}$. RS continues to perform energy harvesting; the amount of harvested energy is the same as for Mode I.

(16) $$E_{H}^{DF,II}=\eta TP_{BS}\left|h_{br}\right|{}^{2}.$$

In Mode III, RS has not only accumulated sufficient energy but also decoded the composited signal successfully. So, it re-codes the signal and re-transmits the combined signal $x\left(t_{2}\right)=a_{p}x_{p}+a_{s}x_{s}$ to both SR and PR. Thus, in the second phase, the signals received by SR and PR are expressed by the following:

(17) $$y_{p}^{DF}\left(t_{2}\right)=\sqrt{P_{RS}}h_{rp}\left(a_{p}x_{p}+a_{s}x_{s}\right)+n_{rp},$$

(18) $$y_{s}^{DF}\left(t_{2}\right)=\sqrt{P_{RS}}h_{rs}\left(a_{p}x_{p}+a_{s}x_{s}\right)+n_{rs},$$

where $n_{rp}∼CN\left(0,\delta^{2}\right)andn_{rs}∼CN\left(0,\delta^{2}\right)$ represent the received AWGN of PR and SR, respectively.

We suppose that both PR and SR can obtain their own information by canceling the information, which is received in the first transmission phase and belongs to other receivers. Hence, the SINR at PR and SR are given by the following:

(19) $$\gamma_{rp}^{DF}=\frac{a_{p}^{2}P_{RS}{\left|h_{rp}\right|}^{2}}{\delta^{2}},$$

(20) $$\gamma_{rs}^{DF}=\frac{a_{s}^{2}P_{RS}{\left|h_{rs}\right|}^{2}}{\delta^{2}},$$

respectively.

## 3. Outage Performance Analysis with AF Relaying Scheme

### 3.1. Energy Accumulation with AF Relaying Scheme

This energy accumulation scheme adopts a discrete-time energy harvesting model [24]. We suppose that the battery capacity of RS is $E_{C}$ and discretizing into L+1 levels. Let the discretization level be represented as $E_{l}\left(l=0,1,\cdots ,L\right)$, which can be defined by the following:

(21) $$\begin{array}{cc} \; & E_{l}=\left[E_{l},E_{l+1}\right),0\leq l\leq L, \end{array}$$

where $E_{l}=\frac{lE_{C}}{L}$ is *l*th energy level of the battery.

The charging and discharging behavior of RS’s battery can be regarded as a stochastic process with discrete time, where we can use a Markov chain (MC) with L+1 states to model. In a MC, the conditional probability distribution of the next state is only affected by the current state; in other words, it has the memorylessness of stochastic processes in probability theory and statistics, which is also called the Markov property [24]. Therefore, we detail the state transition probabilities of the AF scheme. In our notations, $S_{l}$ is the status of RS’s current energy level, and $P_{i,j}^{AF}$ denotes the probability of transition from state $S_{i}$ to state $S_{j}$ in the AF relaying scheme.

#### 3.1.1. $S_{l}\to S_{l},0\leq l<L$

The energy level of RS’s battery remains unchanged only when the amount of energy harvested in Mode I is less than $\frac{E_{c}}{L}$. The corresponding transition probability is expressed as follows:

(22) $$\begin{array}{cc} P_{l,l}^{AF} & =\mathrm{Pr}\left\{E_{H}^{AF}<\frac{E_{C}}{L}\right\}=F_{\left|h_{br}\right|{}^{2}}\left(\frac{E_{C}}{\eta LP_{BS}}\right)\\ & =1-\exp\left(-\frac{E_{C}}{\eta L\lambda_{br}P_{BS}}\right). \end{array}$$

#### 3.1.2. $S_{l}\to S_{m},0\leq l<m<L$

The energy level of the battery turns to level *m*, that is, the amount of energy harvested in Mode I is between $E_{m-l}$ and $E_{m-l+1}$. The transition probability of this case can be given by the following:

(23) $$\begin{array}{cc} P_{l,m}^{AF} & =\mathrm{Pr}\left\{\frac{E_{C}\left(m-l\right)}{L}\leq E_{H}^{AF}<\frac{E_{C}\left(m-l+1\right)}{L}\right\}\\ \; & =F_{\left|h_{br}\right|{}^{2}}\left(\frac{E_{C}\left(m-l+1\right)}{\eta LP_{BS}}\right)-F_{\left|h_{br}\right|{}^{2}}\left(\frac{E_{C}\left(m-l\right)}{\eta LP_{BS}}\right). \end{array}$$

#### 3.1.3. $S_{l}\to S_{L},0\leq l<L$

The battery becomes fully charged during Mode I, and this corresponding transition probability can be expressed by the following:

(24) $$\begin{array}{cc} P_{l,L}^{AF} & =\mathrm{Pr}\left\{E_{H}^{AF}\geq \frac{E_{C}\left(L-l\right)}{L}\right\}\\ \; & =1-\mathrm{Pr}\left\{E_{H}^{AF}<\frac{E_{C}\left(L-l\right)}{L}\right\}\\ \; & =1-F_{\left|h_{br}\right|{}^{2}}\left(\frac{E_{C}\left(L-l\right)}{\eta LP_{BS}}\right) \end{array}$$

#### 3.1.4. $S_{m}\to S_{l},0\leq l<m\leq L$

This situation occurs when the RS discharges as an AF relay in Mode II, and the corresponding transition probability is deduced as follows:

(25) $$P_{m,l}^{AF}=\mathrm{Pr}\left\{E_{T}=\frac{E_{C}\left(m-l\right)}{L}\right\}=\left\{\begin{array}{cc} 0, & E_{T}\neq \frac{E_{C}\left(m-l\right)}{L}\\ 1, & E_{T}=\frac{E_{C}\left(m-l\right)}{L} \end{array}\right.$$

According to the analyses above, we can obtain the state transition matrix for the AF relaying scheme, i.e., $P=\left[P_{i,j}^{AF}\right]{}_{\left(L+1)(L+1\right)}$, which is used to calculate the associated steady-state probabilities. First of all, it can be proved that the MC with P is homogeneous and random. In addition, MC is aperiodic and irreducible because each state can be obtained from transition of other states within a limited time and the corresponding transition probability is non-zero. Given the above two points, a unique steady-state probability vector exists, which can be obtained by solving a set of balance equations as follows:

(26) $$\begin{array}{c} \pi ={\left(B+P^{T}-I\right)}^{-1}b \end{array},$$

where $\pi ={\left[\pi_{0},\pi_{1},\cdots ,\pi_{L}\right]}_{1\times (L+1)}^{T}$ and $\sum_{i=0}^{L}\pi_{i}=1$, **I** denotes an identity matrix, **B** represents a matrix with $\forall B_{i,j}=1\left(1\leq i\leq L+1,1\leq j\leq L+1\right)$ and $b={\left(1,1,\text{…},1\right)}^{T}$ [25].

Thus, the probability that the remaining energy of the battery is higher than or equal to $E_{T}$ in AF relaying is defined as follows:

(27) $$P_{e}^{AF}=\sum_{i=l}^{L}\pi_{i},l=\underset{l\in 1,\cdots ,L}{\arg\min}\left\{E_{l}\geq E_{T}\right\}$$

### 3.2. Outage Probability with AF Relaying Scheme

For the discrete time energy accumulation, we respectively analyze the outage probabilities for both primary and secondary systems by adopting the total probability theory. The corresponding outage probabilities can be expressed as follows:

(28) $$P_{out}^{AF}\left(P\right)=P^{AF}\left(A\right)P_{out}^{AF}\left(P_{A}\right)+P^{AF}\left(B\right)P_{out}^{AF}\left(P_{B}\right),$$

(29) $$P_{out}^{AF}\left(S\right)=P^{AF}\left(A\right)P_{out}^{AF}\left(S_{A}\right)+P^{AF}\left(B\right)P_{out}^{AF}\left(S_{B}\right),$$

respectively, where $P^{AF}\left(A\right)=1-P_{e}^{AF}$ and $P^{AF}\left(B\right)=P_{e}^{AF}$ denote the probability of performing Mode I and Mode II in any transmission block. $P_{out}^{AF}\left(P_{A}\right)$ and $P_{out}^{AF}\left(P_{B}\right)$ represent the outage probabilities of the primary system in Mode I and Mode II, respectively, while the outage probabilities of the secondary system in Mode I and Mode II are represented as $P_{out}^{AF}\left(S_{A}\right)$ and $P_{out}^{AF}\left(S_{B}\right)$, respectively.

#### 3.2.1. Outage Probabilities of Primary System

In Mode I, BS conveys the signal to PR without RS. An outage event occurs only if the primary target data rate $r_{p}$ is higher than the achievable rate. Therefore, the outage probability of the primary system is expressed as follows:

(30) $$\begin{array}{cc} P_{out}^{AF}\left(P_{A}\right) & =\mathrm{Pr}\left\{\gamma_{bp}<R_{p}\right\}\\ \; & =\mathrm{Pr}\left\{|h_{bp}{|}^{2}<\frac{R_{p}\delta^{2}}{P_{BS}\left(a_{p}^{2}-a_{s}^{2}R_{p}\right)}\right\}, \end{array}$$

where $R_{P}=2^{r_{p}}-1$.

- If $R_{p}\geq \frac{a_{p}^{2}}{a_{s}^{2}}$,
  (31) $$P_{out}^{AF}\left(P_{A}\right)=1$$
- If $R_{p}<\frac{a_{p}^{2}}{a_{s}^{2}}$,
  (32) $$P_{out}^{AF}\left(P_{A}\right)=\mathrm{Pr}\left\{\left|h_{bp}{|}^{2}<\varphi_{1}\right.\right\}=1-\exp\left(-\frac{\varphi_{1}}{\lambda_{bp}}\right)$$
  where $\varphi_{1}=\frac{R_{p}\delta^{2}}{P_{BS}\left(a_{p}^{2}-a_{s}^{2}R_{p}\right)}=\frac{R_{p}}{\alpha \left(a_{p}^{2}-a_{s}^{2}R_{p}\right)}$.

In Mode II, the energy of the battery is sufficient for RS performing cooperative transmission. An outage occurs when neither the direct transmission nor the cooperative transmission succeeds. Hence, the primary outage probability in Mode II is derived as follows:

- If $R_{p}\geq \frac{a_{p}^{2}}{a_{s}^{2}}$,
  (33) $$P_{out}^{AF}\left(P_{B}\right)=1$$
- If $R_{p}<\frac{a_{p}^{2}}{a_{s}^{2}}$,
  (34) $$\begin{array}{cc} P_{out}^{AF}\left(P_{B}\right)= & \mathrm{Pr}\left\{\max\left(\gamma_{bp}^{},\gamma_{rp}^{AF}\right)<R_{p}\right\}\\ = & \;\left[1\;-\;\exp(\;-\;\frac{\varphi_{1}}{\lambda_{bp}})\right]\;\;\left[1\;-\;\exp\;\left(\;-\varphi_{2}\;\left(\frac{1}{\lambda_{rp}}+\frac{1}{\lambda_{br}}\right)\;\right)\right.\\ \; & \left.\times \sqrt{\frac{4\varphi_{2}\left(1+\alpha \varphi_{2}\right)}{\lambda_{br}\lambda_{rp}\alpha }}K_{1}\left(\sqrt{\frac{4\varphi_{2}\left(1+\alpha \varphi_{2}\right)}{\lambda_{br}\lambda_{rp}\alpha }}\right)\right] \end{array}$$
  where $\varphi_{2}=\frac{R_{p}\delta^{2}}{P_{RS}\left(a_{p}^{2}-a_{s}^{2}R_{p}\right)}=\frac{R_{p}}{\beta \left(a_{p}^{2}-a_{s}^{2}R_{p}\right)}$ and $K_{1}$(·) denotes the first order modified Bessel function with second kind [26].

**Proof.**  Please refer to Appendix A. □

#### 3.2.2. Outage Pobabilities of Secondary System

In Mode I, the BS directly communicates with SR which applies SIC technique and needs to detect the PR’s message first. The transmission is success when the primary target data rate $r_{p}$ is lower than the achievable rates for PR’s message and the achievable rate for SR’s message is larger than the secondary target data rate $r_{s}$. Hence, the outage probability of the secondary system can be formulated as follows:

(35) $$\begin{array}{cc} P_{out}^{AF}\left(S_{A}\right) & =1-\mathrm{Pr}\{\gamma_{bs1}^{}\geq R_{p},\gamma_{bs2}^{}\geq R_{s}\}\;\\ \; & =1-\mathrm{Pr}\left\{{\left|h_{bs}\right|}^{2}\geq \varphi_{1},{\left|h_{bs}\right|}^{2}\geq \phi_{1}\right\}\;\\ \; & =1-\mathrm{Pr}\left\{{\left|h_{bs}\right|}^{2}\geq \max\left(\varphi_{1},\phi_{1}\right)\right\}\;\\ \; & =1-\exp\left(-\frac{\theta_{1}}{\lambda_{bs}}\right), \end{array}$$

where $\phi_{1}=\frac{R_{s}\delta^{2}}{P_{BS}{a_{s}}^{2}}=\frac{R_{s}}{\alpha {a_{s}}^{2}}$, $\theta_{1}=\max\left(\varphi_{1},\phi_{1}\right)$, and $R_{S}=2^{r_{s}}-1$.

In Mode II, similar to the analysis of the primary system, the outage probability of the secondary system is given as follows:

(36) $$\begin{array}{cc} P_{out}^{AF}\left(S_{B}\right)= & \left(1-\mathrm{Pr}\{\gamma_{bs1}^{}\geq R_{p},\gamma_{bs2}^{}\geq R_{s}\}\right)\\ \; & \times \left(1-\mathrm{Pr}\{\gamma_{rs1}^{AF}\geq R_{p},\gamma_{rs2}^{AF}\geq R_{s}\}\right)\\ = & \left\{\begin{array}{c} \left(1-\exp\left(-\frac{\theta_{1}}{\lambda_{bp}}\right)\right)\left[1-\int_{\varphi_{2}}^{\phi_{2}}\frac{1}{\lambda_{rp}}\exp\left(-\frac{x}{\lambda_{rp}}-\frac{\varphi_{2}\left(1+\alpha x\right)}{\lambda_{br}\alpha \left(x-\varphi_{2}\right)}\right)dx\right],\;\\ if\;\;R_{s}\geq \beta {\left|h_{rs}\right|}^{2}a_{s}^{2}\;\;and\;\;R_{p}<\frac{a_{p}^{2}\beta {\left|h_{rs}\right|}^{2}}{a_{s}^{2}\beta {\left|h_{rs}\right|}^{2}+1}<\frac{a_{p}^{2}}{a_{s}^{2}};\\ 1-\exp\left(-\frac{\theta_{1}}{\lambda_{bp}}\right),\\ if\;\;R_{s}\geq \beta {\left|h_{rs}\right|}^{2}a_{s}^{2}\;\;and\;\;R_{p}\geq \frac{a_{p}^{2}\beta {\left|h_{rs}\right|}^{2}}{a_{s}^{2}\beta {\left|h_{rs}\right|}^{2}+1};\;\\ \left(1-\exp\left(-\frac{\theta_{1}}{\lambda_{bp}}\right)\right)\left[1-\exp\left(-\theta_{2}\left(\frac{1}{\lambda_{rp}}+\frac{1}{\lambda_{br}}\right)\right)\right.\left.\sqrt{\frac{4\theta_{2}\left(1+\alpha \theta_{2}\right)}{\lambda_{br}\lambda_{rp}\alpha }}K_{1}\left(\sqrt{\frac{4\theta_{2}\left(1+\alpha \theta_{2}\right)}{\lambda_{br}\lambda_{rp}\alpha }}\right)\right],\;\\ if\;\;R_{s}<\beta {\left|h_{rs}\right|}^{2}a_{s}^{2}\;\;and\;\;R_{p}<\frac{a_{p}^{2}\beta {\left|h_{rs}\right|}^{2}}{a_{s}^{2}\beta {\left|h_{rs}\right|}^{2}+1}<\frac{a_{p}^{2}}{a_{s}^{2}};\;\\ \left(1-\exp\left(-\frac{\theta_{1}}{\lambda_{bp}}\right)\right)\left[1-\int_{\phi_{2}}^{\varphi_{2}}\frac{1}{\lambda_{rp}}\exp\left(-\frac{x}{\lambda_{rp}}-\frac{\varphi_{2}\left(1+\alpha x\right)}{\lambda_{br}\alpha \left(x-\varphi_{2}\right)}\right)dx\right],\;\\ if\;\;R_{s}<\beta {\left|h_{rs}\right|}^{2}a_{s}^{2}\;\;and\;\;R_{p}\geq \frac{a_{p}^{2}\beta {\left|h_{rs}\right|}^{2}}{a_{s}^{2}\beta {\left|h_{rs}\right|}^{2}+1}\leq R_{p}\leq \frac{a_{p}^{2}}{a_{s}^{2}};\\ 1-\exp\left(-\frac{\theta_{1}}{\lambda_{bp}}\right),\\ if\;\;R_{s}<\beta {\left|h_{rs}\right|}^{2}a_{s}^{2}\;\;and\;\;R_{p}\geq \frac{a_{p}^{2}}{a_{s}^{2}}; \end{array}\right. \end{array}$$

where $\theta_{2}=\max\left(\varphi_{2},\phi_{2}\right)$.

**Proof.**  Please refer to Appendix B. □

## 4. Outage Performance with DF Relaying Scheme

### 4.1. Energy Accumulation with DF Relaying Scheme

In the situation of DF relaying, we use the same discrete-time energy harvesting model [24] as the AF scheme. The state transition probabilities of DF scheme is further discussed in detail in the sequel. Let $S_{l}$ denote RS’s current energy level, and let $P_{i,j}^{DF}$ represent the transition probability from state $S_{i}$ to state $S_{j}$ in the DF scheme.

#### 4.1.1. $S_{0}\to S_{0}$

This situation means that the empty battery performs energy harvesting in Mode I, and the amount of harvested energy is less than $\frac{E_{C}}{L}$. Hence, the corresponding transition probability is derived as follows:

(37) $$\begin{array}{cc} P_{0,0}^{DF} & =\mathrm{Pr}\left\{E_{H}^{DF,I}<\frac{E_{C}}{L}\right\}=F_{{\left|h_{br}\right|}^{2}}\left(\frac{E_{C}}{L\eta P_{BS}}\right)\\ \; & =1-\exp(-\frac{E_{C}}{L\eta P_{BS}\lambda_{br}}) \end{array}$$

#### 4.1.2. $S_{0}\to S_{l}\left(0<l<L\right)$

The empty battery becomes partially charged in Mode I, and the energy level of battery is between $E_{l}$ and $E_{l+1}$. The transition probability of this case can be expressed by the following:

(38) $$\begin{array}{cc} P_{0,l}^{DF} & =\mathrm{Pr}\left\{\frac{lE_{C}}{L}<E_{H}^{\mathrm{DF},I}<\frac{\left(l+1\right)E_{C}}{L}\right\}\\ \; & =F_{{\left|h_{br}\right|}^{2}}\left(\frac{\left(l+1\right)E_{C}}{L\eta P_{BS}}\right)-F_{{\left|h_{br}\right|}^{2}}\left(\frac{lE_{C}}{L\eta P_{BS}}\right) \end{array}$$

#### 4.1.3. $S_{0}\to S_{L}$

The case that an empty battery is charged to full is similar to the corresponding situation of AF scheme. Hence, the transition probability is evaluated as follows:

(39) $$P_{0,L}^{DF}=\mathrm{Pr}\left\{E_{H}^{\mathrm{DF},I}\geq E_{C}\right\}=1-F_{{\left|h_{br}\right|}^{2}}\left(\frac{E_{C}}{\eta P_{BS}}\right)$$

#### 4.1.4. $S_{l}\to S_{l}\left(0<l<L\right)$

The status of the battery with non-empty but not full energy is unchanged in either Mode I or Mode II. RS’s battery keeps harvesting energy but the harvested energy is less than $\frac{E_{C}}{L}$. The transition probability is given as follows:

(40) $$\begin{array}{c} P_{l,l}^{DF}=\mathrm{Pr}\left\{\left[\left(E_{T}>\frac{lE_{C}}{L}\right)⋂\left(E_{H}^{\mathrm{DF},I}<\frac{E_{C}}{L}\right)\right]\right.⋃\left[{\left(E_{T}\leq \frac{lE_{C}}{L}\right)}_{}\right.\;\\ \begin{array}{cc} \; & \; \end{array}⋂\left.\left.{\left(E_{H}^{\mathrm{DF},\mathrm{II}}<\frac{E_{C}}{L}\right)⋂\left(\left(\gamma_{br1}<R_{P}\right)⋃\left(\left(\gamma_{br1}\geq R_{P}\right)⋂\left.\left(\gamma_{br2}<R_{s}\right)\right)\right.\right)}_{}\right]\right\}\;\\ =\left\{\begin{array}{c} F_{{\left|h_{br}\right|}^{2}}\left(\frac{E_{C}}{L\eta P_{BS}}\right),if\;\;E_{T}>\frac{lE_{C}}{L};\;\\ F_{{\left|h_{br}\right|}^{2}}\left(\varphi_{1}\right)F_{{\left|h_{br}\right|}^{2}}\left(\phi_{1}\right)-{F_{{\left|h_{br}\right|}^{2}}}^{2}\left(\varphi_{1}\right),\;\\ \begin{array}{cccccc} \; & \; & & \; & & \; \end{array}if\;\;E_{T}\leq \frac{lE_{C}}{L}\;\;and\;\;R_{P}\leq \frac{a_{1}}{c+a_{2}},R_{s}\leq \frac{a_{2}}{c};\;\\ F_{{\left|h_{br}\right|}^{2}}\left(\varphi_{1}\right)F_{{\left|h_{br}\right|}^{2}}\left(\frac{E_{C}}{L\eta P_{BS}}\right)-{F_{{\left|h_{br}\right|}^{2}}}^{2}\left(\varphi_{1}\right),\;\\ \begin{array}{cccccc} \; & \; & & \; & & \; \end{array}if\;\;E_{T}\leq \frac{lE_{C}}{L}\;\;and\;\;R_{P}\leq \frac{a_{1}}{c+a_{2}},R_{s}>\frac{a_{2}}{c};\;\\ F_{{\left|h_{br}\right|}^{2}}\left(\frac{E_{C}}{L\eta P_{BS}}\right)F_{{\left|h_{br}\right|}^{2}}\left(\phi_{1}\right)-F_{{\left|h_{br}\right|}^{2}}\left(\frac{E_{C}}{L\eta P_{BS}}\right)F_{{\left|h_{br}\right|}^{2}}\left(\varphi_{1}\right),\;\\ \begin{array}{cccccc} \; & \; & & \; & & \; \end{array}if\;\;E_{T}\leq \frac{lE_{C}}{L}\;\;and\;\;\frac{a_{1}}{c+a_{2}}\leq R_{P}\leq \frac{a_{p}^{2}}{a_{S}^{2}},R_{s}\leq \frac{a_{2}}{c};\;\\ {F_{{\left|h_{br}\right|}^{2}}}^{2}\left(\frac{E_{C}}{L\eta P_{BS}}\right)-F_{{\left|h_{br}\right|}^{2}}\left(\frac{E_{C}}{L\eta P_{BS}}\right)F_{{\left|h_{br}\right|}^{2}}\left(\varphi_{1}\right),\;\\ \begin{array}{cccccc} \; & \; & & \; & & \; \end{array}if\;\;E_{T}\leq \frac{lE_{C}}{L}\;\;and\;\;\frac{a_{1}}{c+a_{2}}\leq R_{P}\leq \frac{a_{p}^{2}}{a_{S}^{2}},R_{s}>\frac{a_{2}}{c};\;\\ 0,\begin{array}{ccccc} \; & \; & & \; & \end{array}ifE_{T}\leq \frac{lE_{C}}{L}andR_{P}>\frac{a_{p}^{2}}{a_{S}^{2}}.\; \end{array}\right. \end{array}$$

where $a_{1}=E_{c}a_{p}^{2}$, $a_{2}=E_{c}a_{p}^{2}$, $c=L\eta \delta^{2}$. The derived process of (40) is similar to (36) and omitted.

#### 4.1.5. $S_{l}\to S_{m}\left(0<l<m<L\right)$

The amount of energy harvested by the non-empty battery in Mode I or Mode II is between $E_{m-l}$ and $E_{m-l+1}$. At this time, the non-empty battery’s energy level turns to level *m* and the corresponding conversion probability can be derived as follows:

(41) $$\begin{array}{c} P_{l,m}^{DF}=\mathrm{Pr}\left\{\left[\left(E_{T}>\frac{lE_{C}}{L}\right)⋂\left(\frac{\left(m-l\right)E_{C}}{L}\leq E_{H}^{\mathrm{DF},I}<\frac{\left(m-l+1\right)E_{C}}{L}\right)\right]⋃\left[\left(E_{T}\leq \frac{lE_{C}}{L}\right)\right.\right.\;\\ \begin{array}{cc} \; & \; \end{array}\left.\left.⋂\left(\frac{\left(m-l\right)E_{C}}{L}\leq E_{H}^{\mathrm{DF},\mathrm{II}}<\frac{\left(m-l+1\right)E_{C}}{L}\right)⋂\left(\left(\gamma_{br1}<R_{P}\right)⋃\left(\left(\gamma_{br1}\geq R_{P}\right)⋂\left.\left(\gamma_{br2}<R_{s}\right)\right)\right.\right)\right]\right\}\;\\ \begin{array}{c} \; \end{array}=\left\{\begin{array}{c} F_{{\left|h_{br}\right|}^{2}}\left(\frac{\left(m-l+1\right)E_{C}}{L\eta P_{BS}}\right)-F_{{\left|h_{br}\right|}^{2}}\left(\frac{\left(m-l\right)E_{C}}{L\eta P_{BS}}\right),if\;\;E_{T}>\frac{lE_{C}}{L};\;\\ 0,\begin{array}{c} \; \end{array}if\;\;E_{T}\leq \frac{lE_{C}}{L}\;\;and\;\;R_{P}<\frac{\left(m-l\right)a_{1}}{c+\left(m-l\right)a_{2}};\;\\ 0,\begin{array}{c} \; \end{array}ifE_{T}\leq \frac{lE_{C}}{L}and\frac{\left(m-l\right)a_{1}}{c+\left(m-l\right)a_{2}}\leq R_{P}\leq \frac{\left(m-l+1\right)a_{1}}{c+\left(m-l+1\right)a_{2}},R_{s}<\frac{\left(m-l\right)a_{2}}{c};\;\\ \left[F_{{\left|h_{br}\right|}^{2}}\left(\varphi_{1}\right)-F_{{\left|h_{br}\right|}^{2}}\left(\frac{\left(m-l\right)E_{C}}{L\eta P_{BS}}\right)\right]\left[F_{{\left|h_{br}\right|}^{2}}\left(\phi_{1}\right)-F_{{\left|h_{br}\right|}^{2}}\left(\varphi_{1}\right)\right],\;\\ \begin{array}{cc} & \; \end{array}if\;\;E_{T}\leq \frac{lE_{C}}{L}\;\;and\;\;\frac{\left(m-l\right)a_{1}}{c+\left(m-l\right)a_{2}}\leq R_{P}\leq \frac{\left(m-l+1\right)a_{1}}{c+\left(m-l+1\right)a_{2}},\frac{\left(m-l\right)a_{2}}{c}\leq R_{s}\leq \frac{\left(m-l+1\right)a_{2}}{c};\;\\ \left[F_{{\left|h_{br}\right|}^{2}}\left(\varphi_{1}\right)-F_{{\left|h_{br}\right|}^{2}}\left(\frac{\left(m-l\right)E_{C}}{L\eta P_{BS}}\right)\right]\left[F_{{\left|h_{br}\right|}^{2}}\left(\frac{\left(m-l+1\right)E_{C}}{L\eta P_{BS}}\right)-F_{{\left|h_{br}\right|}^{2}}\left(\varphi_{1}\right)\right]\;\\ \begin{array}{cc} & \; \end{array}if\;\;E_{T}\leq \frac{lE_{C}}{L}\;\;and\;\;\frac{\left(m-l\right)a_{1}}{c+\left(m-l\right)a_{2}}\leq R_{P}\leq \frac{\left(m-l+1\right)a_{1}}{c+\left(m-l+1\right)a_{2}},R_{s}>\frac{\left(m-l+1\right)a_{2}}{c};\;\\ 0,if\;\;E_{T}\leq \frac{lE_{C}}{L}\;\;and\;\;\frac{\left(m-l+1\right)a_{1}}{c+\left(m-l+1\right)a_{2}}<R_{P}<\frac{a_{p}^{2}}{a_{S}^{2}}. \end{array}\right. \end{array}$$

#### 4.1.6. $S_{l}\to S_{L}$

In Mode I or Mode II, when the amount of harvested energy is equal to or exceeds the battery’s upper limit capacity, the battery will be fully charged. Hence, the transition probability can be written as follows:

(42) $$\begin{array}{c} P_{l,L}^{DF}=\mathrm{Pr}\left\{\;\left[\left(E_{T}>\frac{lE_{C}}{L}\right)⋂\left(E_{H}^{\mathrm{DF},I}\geq \frac{\left(L-l\right)E_{C}}{L}\right)\right]\right.\;\\ \begin{array}{cc} \; & \; \end{array}\begin{array}{c} \;⋃[\left(E_{T}\leq \frac{lE_{C}}{L}\right)⋂\left(E_{H}^{\mathrm{DF},\mathrm{II}}\geq \frac{\left(L-l\right)E_{C}}{L}\right)\;\\ \;\left.⋂\;\left(\;\left(\gamma_{br}^{x_{p}}<R_{P}\right)⋃\;\left(\;\left(\gamma_{br}^{x_{p}}\geq R_{P}\right)⋂\left.\left(\gamma_{br}^{x_{s}}<R_{s}\right)\right)\right.\;\right)\;]\;\right\} \end{array}\;\\ =\left\{\begin{array}{c} \;\;1-F_{{\left|h_{br}\right|}^{2}}\left(\frac{\left(L-l\right)E_{C}}{L\eta P_{BS}}\right),ifE_{T}>\frac{lE_{C}}{L};\;\\ \;\;0,if\;\;E_{T}\leq \frac{lE_{C}}{L}\;\;and\;\;R_{P}<\frac{\left(L-l\right)a_{1}}{c+\left(L-l\right)a_{2}}<\frac{a_{p}^{2}}{a_{S}^{2}};\;\\ \;\;0,if\;\;E_{T}\leq \frac{lE_{C}}{L}\;\;and\;\;\frac{\;\left(\;L-l\;\right)a_{1}}{c+\;\left(\;L-l\;\right)a_{2}}\leq R_{P}\;<\;\frac{a_{p}^{2}}{a_{S}^{2}},R_{s}\;\leq \;\frac{\;\left(\;L\;-\;l\;\right)a_{2}}{c};\;\\ \;\;\left[\;F_{{\;\left|h_{br}\;\right|}^{2}}\;\left(\;\varphi_{1}\;\right)\;-\;F_{{\left|h_{br}\right|}^{2}}\;\left(\;\frac{\;\left(\;L\;-\;l\right)\;E_{C}}{L\eta P_{BS}}\;\right)\;\right]\;\;\left[\;F_{{\left|h_{br}\right|}^{2}}\;\left(\;\phi_{1}\;\right)\;-\;F_{{\left|h_{br}\right|}^{2}}\;\left(\;\varphi_{1}\;\right)\;\right]\;,\;\\ \begin{array}{cc} & \; \end{array}\;\;if\;\;E_{T}\;\leq \;\frac{lE_{C}}{L}\;\;and\;\;\frac{\;\left(\;L\;-\;l\right)a_{1}}{c+\;\left(\;L\;-l\right)a_{2}}\;\leq \;R_{p}\;<\;\frac{a_{p}^{2}}{a_{S}^{2}},R_{s}\;>\;\frac{\;\left(\;L\;-\;l\right)a_{2}}{c};\;\\ \;\;0,if\;\;E_{T}\leq \frac{lE_{C}}{L}\;\;and\;\;R_{P}\geq \frac{a_{p}^{2}}{a_{S}^{2}}. \end{array}\right. \end{array}$$

#### 4.1.7. $S_{L}\to S_{L}$

The RS with fully charged battery can perform DF relaying, but due to the wrong decoding of the superposed signal in Mode II, it can only continue to perform energy harvesting. In this case, the value of the harvested energy can be ignored because the energy level of the battery has reached the limit. Therefore, the state transition probability can be derived as follows:

(43) $$\begin{array}{cc} P_{L,L}^{DF} & =\mathrm{Pr}\;\left\{\;\left(\gamma_{br1}\;<\;R_{P}\right)⋃\left[\left(\gamma_{br1}\;\geq \;R_{P}\right)⋂\left(\gamma_{br2}\;<\;R_{s}\right)\right]\;\right\}\\ \; & =F_{{\left|h_{br}\right|}^{2}}\left(\phi_{1}\right) \end{array}$$

#### 4.1.8. $S_{m}\to S_{l}$

The situation will only occur in Mode III, and its corresponding transition probability can be evaluated as follows:

(44) $$\begin{array}{c} P_{m,l}^{DF}=\mathrm{Pr}\left\{\left(\gamma_{br}^{x_{p}}\geq R_{P}\right)⋂\left(\gamma_{br}^{x_{s}}\geq R_{s}\right)⋂\left(E_{T}=\frac{\left(m-l\right)E_{C}}{L}\right)\right\}\;\\ =\left\{\begin{array}{c} \exp\left(-\frac{\theta_{1}}{\lambda_{br}}\right),ifE_{T}=\frac{\left(m-l\right)E_{C}}{L};\;\\ 0,ifE_{T}\neq \frac{\left(m-l\right)E_{C}}{L}. \end{array}\right. \end{array}$$

Let $Q=\left[P_{i,j}^{DF}\right]{}_{\left(L+1)(L+1\right)}$ represent matrix of state transition in DF scheme. A unique steady-state probability vector also can be obtained by solving a set of balance equations as follows:

(45) $$\begin{array}{c} \pi^{\prime }={\left(B+Q^{T}-I\right)}^{-1}b \end{array},$$

where $\pi^{\prime }={\left[\pi_{0}^{\prime },\pi_{1}^{\prime },\cdots ,\pi_{L}^{\prime }\right]}_{1\times (L+1)}^{T}$ and $\sum_{i=0}^{L}\pi_{i}^{\prime }=1$.

Likewise, the probability that remaining energy of RS’s battery exceeds or equals to $E_{T}$ in DF relaying scheme can be illustrated as follows:

(46) $$P_{e}^{DF}=\sum_{i=l}^{L}\pi_{i}^{\prime },l=\underset{l\in 1,\cdots ,L}{\arg\min}\left\{E_{l}\geq E_{T}\right\}.$$

### 4.2. Outage Probability with Non-Linear EH in DF Relaying

Occurrence probabilities of Mode I, Mode II, and Mode III in any transmission block are respectively illustrated as $P^{DF}\left(A\right)$, $P^{DF}\left(B\right)$, and $P^{DF}\left(C\right)$. $P_{out}^{DF}\left(P_{A}\right)$, $P_{out}^{DF}\left(P_{B}\right)$, and $P_{out}^{DF}\left(P_{C}\right)$ are the outage probabilities of the primary system in Mode I, Mode II and Mode III, respectively, while $P_{out}^{DF}\left(S_{A}\right)$, $P_{out}^{DF}\left(S_{B}\right)$ and $P_{out}^{DF}\left(S_{C}\right)$ represent the secondary system’s outage probabilities of Mode I, Mode II, and Mode III, respectively. Therefore, the outage probabilities of the primary system and secondary system are expressed by the following:

(47) $$\begin{array}{cc} P_{out}^{DF}\left(P\right)= & P^{DF}\left(A\right)P_{out}^{DF}\left(P_{A}\right)+P^{DF}\left(B\right)P_{out}^{DF}\left(P_{B}\right)\\ \; & +P^{DF}\left(C\right)P_{out}^{DF}\left(P_{C}\right)\\ P_{out}^{DF}\left(S\right)= & P^{DF}\left(A\right)P_{out}^{DF}\left(S_{A}\right)+P^{DF}\left(B\right)P_{out}^{DF}\left(S_{B}\right)\\ \; & +P^{DF}\left(C\right)P_{out}^{DF}\left(S_{C}\right) \end{array}$$

It can be seen from (27) that the probability of occurrence of Mode I is equivalent to the probability that the energy of RS is less than the output threshold $E_{T}$. Therefore, the probability can be expressed as follows:

(48) $$P^{DF}\left(A\right)=1-P_{e}^{DF}.$$

The system performs Mode II when RS has sufficient energy for the relay but decodes the superimposed signal incorrectly. Thus, the occurrence probability of Mode II can be derived as follows:

(49) $$\begin{array}{cc} P^{DF}\;\left(B\right)\;\; & \;=\;P_{e}^{DF}\;\mathrm{Pr}\;\left\{\;\left(\gamma_{br1}\;<\;R_{p}\right)\cup (\;\;\left(\gamma_{br1}\;\geq \;R_{p}\right)\cap \left.\;\left(\gamma_{br2}\;<\;R_{s}\right)\;)\;\right.\right\}\\ \; & =P_{e}^{DF}F_{{\left|h_{br}\right|}^{2}}\left(\phi_{1}\right) \end{array}$$

When the RS has sufficient energy and decodes the synthetic signal correctly, Mode III is performed. The probability of occurrence of Mode III is obtained as follows:

(50) $$\begin{array}{cc} P^{DF}\left(C\right) & =P_{e}^{DF}\mathrm{Pr}\left\{\gamma_{br1}\geq R_{P},\gamma_{br2}\geq R_{s}\right\}\\ \; & =P_{e}^{DF}\exp\left(-\frac{\theta_{1}}{\lambda_{br}}\right) \end{array}$$

#### 4.2.1. Outage Probability of Primary Probability

Since Mode I of the DF relaying scheme is the same as Mode I of the AF relaying scheme, the primary outage probability of Mode I under the DF relaying scheme outage is equal to that of Mode I under the AF relaying scheme (30), which is expressed as follows:

(51) $$\begin{array}{c} P_{out}^{DF}\left(P_{A}\right)\;=\;\mathrm{Pr}\left\{\gamma_{bp}^{}\;<\;R_{p}\right\}\;=\;\mathrm{Pr}\;\left\{\;|h_{bp}{|}^{2}\;<\;\frac{R_{p}\delta^{2}}{P_{BS}\;\left({a_{p}}^{2}-{a_{s}}^{2}R_{p}\right)\;}\;\right\} \end{array}$$

- If $R_{p}\geq \frac{{a_{p}}^{2}}{{a_{s}}^{2}}$,
  (52) $$P_{out}^{DF}\left(P_{A}\right)=1$$
- If $R_{p}<\frac{{a_{p}}^{2}}{{a_{s}}^{2}}$,
  (53) $$P_{out}^{AF}\left(P_{A}\right)=\mathrm{Pr}\left\{\gamma_{bp}^{}<R_{p}\right\}=1-\exp\left(-\frac{\varphi_{1}}{\lambda_{bp}}\right)$$

In Mode II, RS decoding fails and BS conveys the signals without the assitance of RS. The process is the same as direct transmission under Mode I. Hence, the primary outage probability of Mode II is equal to the primary outage probability of Mode I, that is, $P_{out}^{DF}\left(P_{B}\right)=P_{out}^{AF}\left(P_{A}\right)$.

In Mode III, if the direct or indirect transmission is unsuccessful, the transmission of the system is interrupted, and its occurrence probability is expressed as follows:

- If $R_{p}\geq \frac{{a_{p}}^{2}}{{a_{s}}^{2}}$,
  (54) $$\begin{array}{cc} P_{out}^{DF}\left(P_{C}\right) & =\mathrm{Pr}\left\{\gamma_{bp}^{}<R_{p}\right\}\mathrm{Pr}\left\{\gamma_{rp}^{DF}<R_{p}\right\}\\ \; & =1\;-\;\exp\left(\;-\frac{R_{p}\delta^{2}}{P_{RS}{a_{p}}^{2}\lambda_{rp}}\right) \end{array}$$
- If $R_{p}<\frac{{a_{p}}^{2}}{{a_{s}}^{2}}$,
  (55) $$\begin{array}{cc} P_{out}^{DF}\left(P_{C}\right) & =\mathrm{Pr}\left\{\gamma_{bp}^{}<R_{p}\right\}\mathrm{Pr}\left\{\gamma_{rp}^{DF}<R_{p}\right\}\\ \; & =\mathrm{Pr}\left\{|h_{bp}{|}^{2}<\varphi_{1}\right\}\mathrm{Pr}\left\{|h_{rp}{|}^{2}<\frac{R_{p}\delta^{2}}{P_{RS}{a_{p}}^{2}}\right\}\\ \; & =\;\left[1\;-\;\exp\left(\;-\frac{\varphi_{1}}{\lambda_{bp}}\right)\;\right]\;\left[1\;-\;\exp\left(\;-\frac{R_{p}\delta^{2}}{P_{RS}{a_{p}}^{2}\lambda_{rp}}\right)\;\right] \end{array}$$

#### 4.2.2. Outage Probability of Secondary Probability

Under the DF relay scheme, the analysis of the secondary outage probability is similar to that of the primary outage system, which can be derived as follows:

(56) $$\begin{array}{cc} P_{out}^{DF}\left(S_{A}\right) & =1-\mathrm{Pr}\{\gamma_{bs1}^{}\geq R_{p},\gamma_{bs2}^{}\geq R_{s}\}\\ \; & =1-\exp\left(-\frac{\theta_{1}}{\lambda_{bs}}\right), \end{array}$$

(57) $$P_{out}^{DF}\left(S_{B}\right)=P_{out}^{DF}\left(S_{A}\right),$$

(58) $$\begin{array}{cc} P_{out}^{DF}\left(S_{C}\right)\;= & \left(1\;-\;\mathrm{Pr}\{\gamma_{bs1}^{}\;\geq \;R_{p},\gamma_{bs2}^{}\;\geq \;R_{s}\}\right)\mathrm{Pr}\left\{\gamma_{rs}^{DF}\;<\;R_{s}\right\}\\ = & \left[1-\mathrm{Pr}\left\{{\left|h_{bs}\right|}^{2}\geq \varphi_{1},{\left|h_{bs}\right|}^{2}\geq \phi_{1}\right\}\right]\\ \; & \times \mathrm{Pr}\left\{{\left|h_{rs}\right|}^{2}<\frac{R_{s}\delta^{2}}{P_{RS}{a_{s}}^{2}}\right\}\\ = & \left[1\;-\;\exp\left(\;-\frac{\theta_{1}}{\lambda_{bs}}\right)\right]\;\left[1\;-\;\exp\left(\;-\frac{R_{s}\delta^{2}}{P_{RS}{a_{s}}^{2}\lambda_{rs}}\right)\;\right]. \end{array}$$

## 5. Numerical and Simulation Results

This section demonstrates the influence of each parameter on the performance of the two transmission schemes through simulation experiments. In the meanwhile, we verify the accuracy of the theoretical expressions derived above. If there is no special instruction, the value of the simulation parameters setting in the system model is shown in the Table 1 as following.

### 5.1. Outage Performance of AF Relaying Scheme

**Figure 4:**
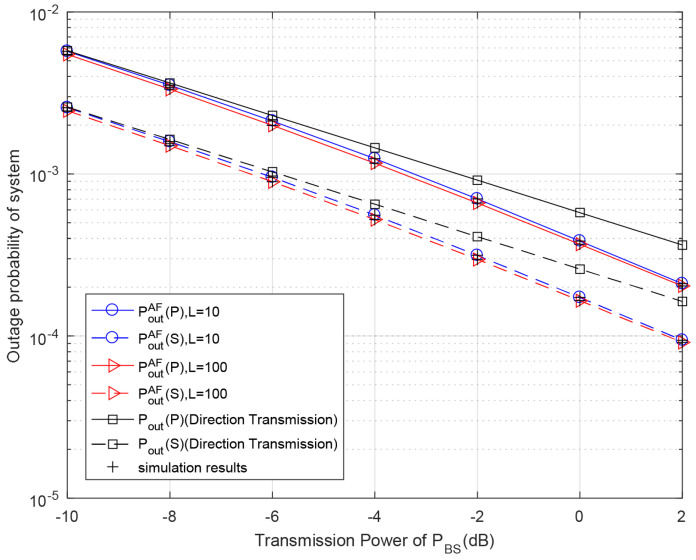
Outage probability with respect to different primary transmission power $P_{BS}$ for different discrete levels of battery capacity in AF relaying scheme. Primary rate $r_{p}=0.5$ bit/s/Hz, secondary rate $r_{s}$ = 0.5 bit/s/Hz, $P_{RS}=P_{BS}$.

Figure 4 demonstrates the outage probabilities of the system with respect to different transmitted power of BS for different discrete levels of battery capacity in the AF relaying protocol. The lower the transmission power of the BS, the less energy RS can harvest. Therefore, the energy of the battery is not enough for performing cooperative transmission, and RS keeps harvesting energy in Mode I. Therefore, when the transmission power of the BS is low, both the primary and secondary outage probabilities are very close to the outage probability of direct transmission scheme. This figure also shows that the outage probabilities of both primary and secondary systems with the proposed AF relaying scheme are lower than the corresponding outage probabilities of the direct transmission scheme. The outage performance of both the primary and secondary systems improves when the numbers of battery levels increases from 10 to 100, which is because more battery levels are helpful for reducing energy waste in the process of energy harvesting. Our theoretical results of primary and secondary systems coincide exactly with the Monte Carlo simulation results.

**Figure 5:**
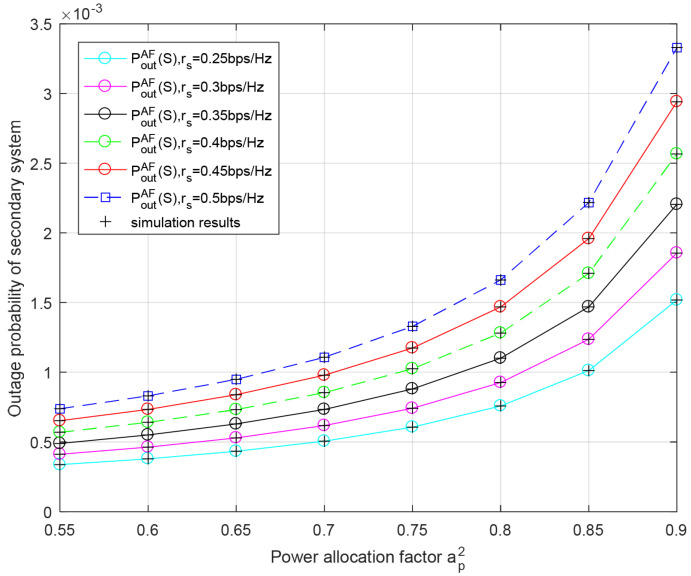
Outage probability of secondary system with respect to power allocation factor $a_{p}^{2}$ for different secondary rate $r_{s}$ in AF relaying scheme. Primary rate $r_{p}$ = 0.25 bit/s/Hz, $P_{RS}=P_{BS}=-10$ dB.

Figure 5 plots the outage probability of the secondary system versus the primary power allocation factor $a_{p}^{2}$ for different transmission rates of the secondary system $r_{s}$ in the AF relaying protocol. It can be seen from this figure that the greater $a_{p}^{2}$ is, the higher the outage probability of the secondary system. This is because as more energy is allocated to transmit data of the primary system, less energy can be used for data transmission of the secondary system. Due to the limited transmission rate supported by the channel, when the value of $a_{p}^{2}$ is fixed, the secondary outage probability increases with the increase in $r_{s}$. The result are consistent with Monte Carlo simulation.

**Figure 6:**
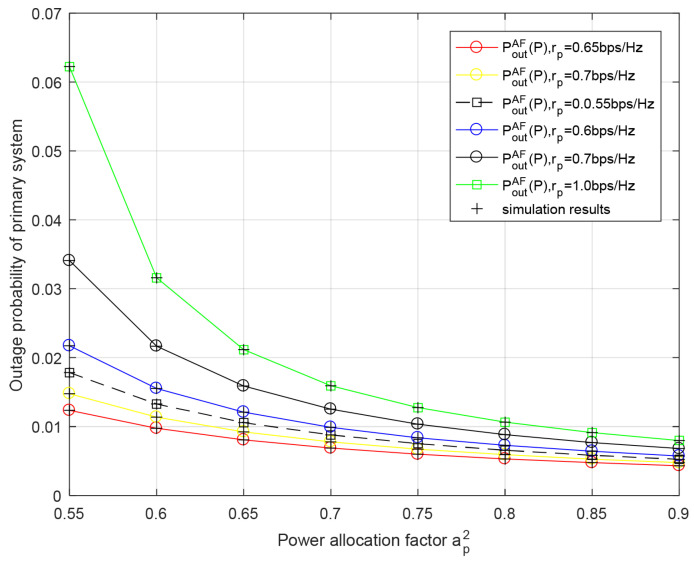
Outage probability of primary system with respect to power allocation factor $a_{p}^{2}$ for different primary rate $r_{p}$ in AF relaying scheme. Secondary rate $r_{s}$ = 0.25 bit/s/Hz, $P_{RS}=P_{BS}=-10$ dB.

Figure 6 demonstrates the outage probability of the primary system versus the power allocation factor $a_{p}^{2}$ for different transmission rates of the primary system $r_{p}$ in the AF relaying protocol. We can observe that the primary system’s outage performance improves with greater $a_{p}^{2}$ since the energy used to convey the primary signal is increased and the interference introduced by transmitting the secondary data is decreased. The theoretical data are basically consistent with the simulation data, which verifies the correctness of the analysis of both the primary outage probability and secondary outage probability.

### 5.2. Outage Performance of DF Relaying Scheme

**Figure 7:**
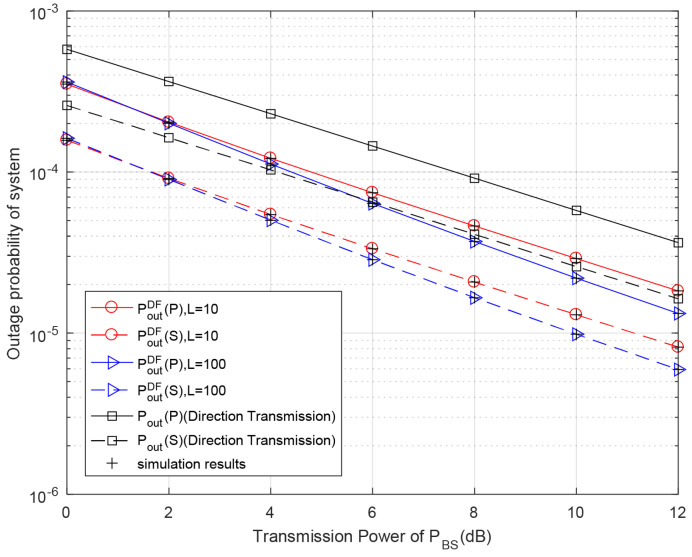
Outage probability with respect to different primary transmission power $P_{BS}$ for different discrete levels of battery capacity in DF relaying scheme. Primary rate $r_{p}$ = 0.5 bit/s/Hz, secondary rate $r_{s}$ = 0.5 bit/s/Hz, $P_{RS}=P_{BS}$.

Figure 7 shows the outage probabilities of both the primary and secondary systems versus the BS’s transmission power for different discrete levels of battery capacity in the proposed DF relaying protocol. For different discrete levels of battery capacity, as $P_{BS}$ increases, the primary outage probabilities under the DF relay scheme gradually decrease and become lower than the outage probabilities under the direct transmission scheme. Similar to the analytical results in Figure 4, the outage performance of both the primary and secondary systems is improved with higher numbers of battery levels. Our theoretical results of primary and secondary systems coincide exactly with the Monte Carlo simulation results.

**Figure 8:**
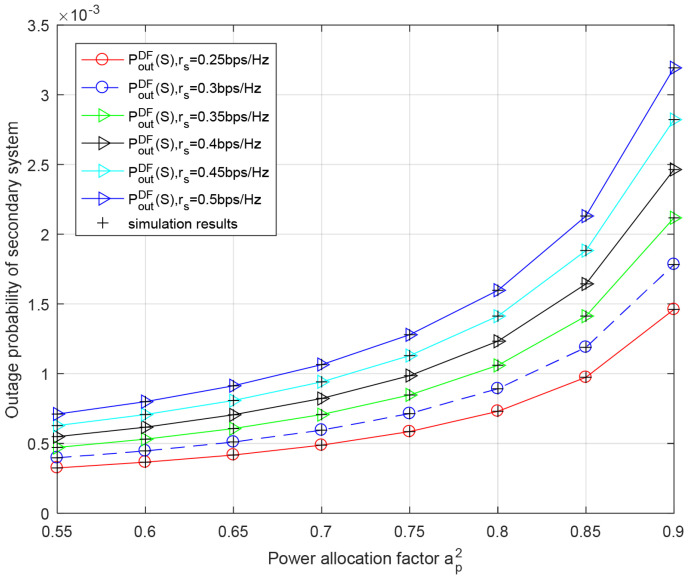
Outage performance of secondary system with respect to power allocation factor $a_{p}^{2}$ for different secondary rate $r_{s}$ in DF relaying scheme. Secondary rate $r_{s}$ = 0.25 bit/s/Hz, $P_{RS}=P_{BS}=-10$ dB.

Figure 8 shows the secondary outage probability versus the power allocation factor $a_{p}^{2}$ for different transmission rates of secondary system $r_{s}$ in the DF relaying scheme. We can see from this figure that as the power allocation factor $a_{p}^{2}$ increases, the outage probability of secondary system gradually becomes higher because less power is used to transmit data of the secondary system. It can also be observed that the outage performance of the secondary system deteriorates with the increase in $r_{s}$.

**Figure 9:**
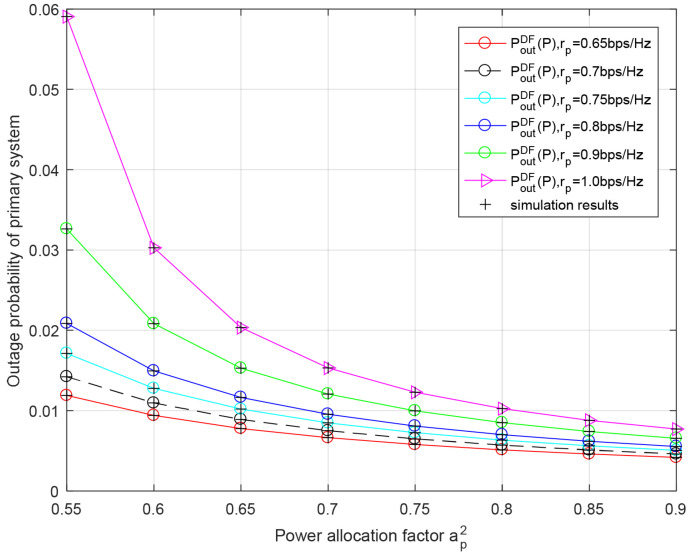
Outage performance of primary system with respect to power allocation factor $a_{p}^{2}$ for different primary rates $r_{p}$ in AF relaying scheme. Secondary rate $r_{s}=0.25$ bit/s/Hz, $P_{RS}=P_{BS}=-10$ dB.

Figure 9 evaluates the primary outage probability versus the power allocation factor $a_{p}^{2}$ for different transmission rates of primary system $r_{p}$ in the DF relaying protocol. From the image, we know that the primary system’s outage performance improves with an increase in $a_{p}^{2}$. This is similar to the situation of the AF relaying scheme. Moreover, the theoretical results are in good agreement with the results of the Monte Carlo simulation.

### 5.3. Comparison of Outage Performance

To demonstrate the advantages of the proposed protocols, we compare two proposed schemes to the scheme of SWIPT-F-NOMA in [21] and the direct transmission scheme in terms of the outage probabilities of both the primary and secondary system. The scheme of SWIPT-F-NOMA in [21] adopts NOMA in cooperation with CRN with SWIPT, which is different from the proposed schemes.

**Figure 10:**
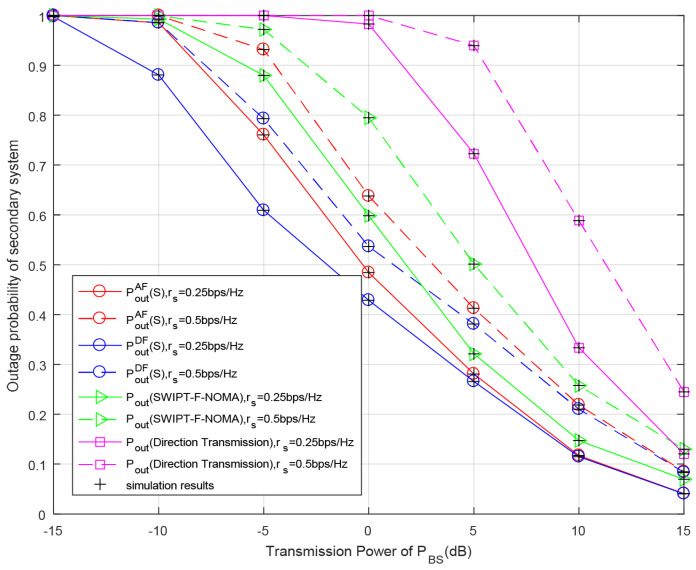
Outage probability of secondary system with respect to different primary transmission power $P_{BS}$ for different secondary rates $r_{s}$. Primary rate $r_{p}$ = 0.5 bit/s/Hz, $P_{RS}=P_{BS}$, the power of noise $\delta^{2}=-10$ dB.

**Figure 11:**
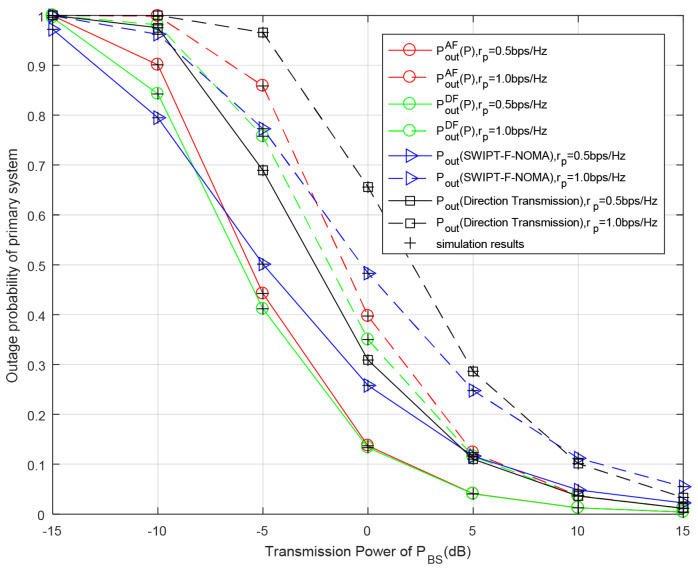
Outage probability of primary system versus primary transmission power $P_{BS}$ for different primary rates $r_{p}$. Secondary rate $r_{s}=0.5$ bit/s/Hz, $P_{RS}=P_{BS}$, the power of noise $\delta^{2}=-10$ dB.

Figure 10 and Figure 11 show the outage probabilities of the whole system with respect to the transmission power of BS $P_{BS}$ for different primary or secondary data rates. We can notice that the outage performance of both the primary and secondary system are continuously improved over the range of the primary transmission power $P_{BS}$ because the larger transmission power $P_{BS}$ means that the required time slots for RS’s battery to accumulate sufficient energy is decreased, so the probability of RS participating in cooperative transmission is increased and the outage probabilities of both the primary and secondary systems are decreased. Similar to the previous analysis, the system outage performance becomes better when the target transmission rate increases. This is because the channel has higher capability to support transmission with the decrease in target rates. As can be seen from Figure 10 and Figure 11, under the conditions of set parameters, the system outage probability of the DF relay scheme is always lower than that of the AF relay scheme, regardless of the primary system or secondary system. In Figure 10, the secondary outage probabilities with the DF- and AF relay schemes are better than those of the SWIPT-F-NOMA scheme and direct transmission. In the direct transmission mode, the RS does not participate in cooperative transmission, so the outage probability of the system is the highest. In Figure 11, when the value of $P_{BS}$ is low, the SWIPT-F-NOMA scheme is superior to the DF and AF relay schemes, but with the increase in $P_{BS}$, the DF and AF relay schemes gradually become superior to the SWIPT-F-NOMA scheme. From these two figures, it can be calculated from the simulation results that when $r_{p}=0.5$ bit/s/Hz and $r_{s}=0.25$ bit/s/Hz, the outage probability of the secondary system in the AF relaying scheme is reduced by 22.2% and 13.4% for $P_{BS}=-5$ dB, compared with the direct transmission and SWIPT-F-NOMA schemes, respectively, while the outage probability of the secondary system in the DF relaying scheme is reduced by 38.3% and 30.6%. When $r_{p}=0.5$ bit/s/Hz and $r_{s}=0.5$ bit/s/Hz, the primary outage probability in the AF relaying scheme is reduced by 37.6% and 14.2% for $P_{BS}=-5$ dB, compared with the direct transmission and SWIPT-F-NOMA schemes, respectively, while the primary outage probabilities in the DF relaying scheme is reduced by 40.5% and 18.7%. Similarly, the analytical results are in good agreement with the simulation results.

## 6. Conclusions

In this paper, we investigate two cooperative transmission schemes by utilizing AF and DF techniques for a wireless-powered CR-NOMA networks, where the battery of relay RS is discretized into several levels. At first, a MC with finite states is used to model the charging and discharging behavior of the battery. In addition, we derive the closed-form expressions of outage probabilities of the primary system and secondary system and verify them through simulation. Finally, we observe from the simulation results that both schemes can greatly improve the outage performance of the overall system and perform better than the relay scheme, using SWIPT and the direct transmission scheme. In particular, the proposed DF relaying protocol outperforms the proposed AF relaying protocol within the range of setting values.

## Figures and Tables

**Table 1:** Lists of Necessary Parameters.

Symbol	Name	Value
$d_{bp}$	Transmit distance from BS to PR	10 m
$d_{bs}$	Transmit distance from BS to SR	5 m
$d_{br}$	Transmit distance from BS to RS	2.5 m
$d_{rp}$	Transmit distance from RS to PR	7.5 m
$d_{rs}$	Transmit distance from RS to SR	2.5 m
$\lambda_{i}$	The means of channel gain	$d_{i}^{-3}$(i=bp,br,bs,rp and rs)
η	Energy conversion efficiency	0.5
$E_{c}$	Total capacity of battery at RS	20 dBm
$E_{T}$	Predefined threshold power at RS	−10 dBm
$\delta^{2}$	AWGNs	−30 dBm
*L*	RS’s battery levels	50
$r_{p}$	Primary target transmission rate	0.65 bps/Hz
$r_{s}$	Secondary target transmission rate	0.25 bps/Hz
$P_{BS}$	transmission power of BS	−10 dBm
$P_{RS}$	transmission power of RS	−10 dBm

## Data Availability

Data are contained within the article.

## Appendix A

**Derivation of (34)**

Mathematically, Equation (34) can be simplified as follows:

(A1) $$\begin{array}{cc} P_{out}^{AF}\left(P_{B}\right) & =\mathrm{Pr}\left\{\max\left(\gamma_{bp},\gamma_{rp}^{AF}\right)<R_{p}\right\}\\ \; & =\mathrm{Pr}\left\{\gamma_{bp}<R_{p}\right\}\mathrm{Pr}\left\{\gamma_{rp}^{AF}<R_{p}\right\} \end{array}$$

Firstly, we consider the first term of (A1), which is same as the primary outage probability in Mode I.

- If $R_{p}\geq \frac{a_{p}^{2}}{a_{s}^{2}}$,
  (A2) $$\mathrm{Pr}\left\{\gamma_{bp}^{}<R_{p}\right\}=1$$
- If $R_{p}<\frac{a_{p}^{2}}{a_{s}^{2}}$,
  (A3) $$\mathrm{Pr}\left\{\gamma_{bp}^{}<R_{p}\right\}=1-\exp\left(\frac{-\varphi_{1}}{\lambda_{bp}}\right)$$

Furthermore, the second term of (A1) can be derived as follows:

(A4) $$\begin{array}{cc} \mathrm{Pr} & \left\{\gamma_{rp}^{AF}<R_{p}\right\}\\ = & \mathrm{Pr}\left\{\frac{\alpha \beta a{{}_{p}^{}}^{2}{\left|h_{rp}\right|}^{2}{\left|h_{br}\right|}^{2}}{\alpha \beta a{{}_{s}^{}}^{2}{\left|h_{rp}\right|}^{2}{\left|h_{br}\right|}^{2}+\alpha \left({\left|h_{rp}\right|}^{2}+{\left|h_{br}\right|}^{2}\right)+1}<R_{p}\right\}\\ = & \mathrm{Pr}\left\{\alpha {\left|h_{br}\right|}^{2}\;\left[\beta {\left|h_{rp}\right|}^{2}\;\left(\;a{{}_{p}^{}}^{2}\;-\;a{{}_{s}^{}}^{2}R_{p}\;\right)\;\;-\;R_{p}\right]\;<\;\alpha R_{p}{\left|h_{rp}\right|}^{2}\;+\;R_{p}\right\} \end{array}$$

- If $R_{p}\geq \frac{a_{p}^{2}}{a_{s}^{2}}$, the following holds:
  (A5) $$\mathrm{Pr}\left\{\gamma_{rp}^{AF}<R_{p}\right\}=1$$
- If $R_{p}<\frac{a_{p}^{2}}{a_{s}^{2}}$, the following holds:
  (A6) $$\begin{array}{cc} \mathrm{Pr}\left\{\gamma_{rp}^{AF}<R_{p}\right\}= & \mathrm{Pr}\left\{{\left|h_{rp}\right|}^{2}\;>\;\varphi_{2},{\left|h_{br}\right|}^{2}\;<\;\frac{\varphi_{2}\left(1\;+\;\alpha {\left|h_{rp}\right|}^{2}\right)}{\alpha ({\left|h_{rp}\right|}^{2}\;-\;\varphi_{2})}\right\}\\ \; & +\mathrm{Pr}\left\{{\left|h_{rp}\right|}^{2}\;\leq \;\varphi_{2}\right\}\\ = & \int_{0}^{\varphi_{2}}\frac{1}{\lambda_{rp}}\exp\left(-\frac{x}{\lambda_{rp}}\right)+\int_{\varphi_{2}}^{\infty }\frac{1}{\lambda_{rp}}\exp\left(-\frac{x}{\lambda_{rp}}\right)\\ \; & \times \int_{0}^{\frac{\varphi_{2}\left(1\;+\;\alpha x\right)}{\alpha (x\;-\;\varphi )}}\frac{1}{\lambda_{br}}\exp\left(\;-\;\frac{y}{\lambda_{br}}\right)dydx\\ = & 1\;+\;\;\int_{\varphi_{2}}^{\infty }\frac{1}{\lambda_{rp}}\exp\;\left(\;-\;\frac{x}{\lambda_{rp}}\;\right)\;\left[\;\left.1\;-\;\exp\left(\;-\;\frac{\varphi_{2}\left(1\;+\;\alpha x\right)}{\lambda_{br}\alpha \left(\;x\;-\varphi \right)}\right)\;\right]\;\;\right.dx\\ \; & -\exp\left(-\frac{\varphi_{2}}{\lambda_{rp}}\right)\\ = & 1-\int_{\varphi_{2}}^{\infty }\frac{1}{\lambda_{rp}}\exp\left(-\frac{x}{\lambda_{rp}}-\frac{\varphi_{2}\left(1+\alpha x\right)}{\lambda_{br}\alpha \left(x-\varphi_{2}\right)}\right)dx \end{array}$$

Applying the principle in [27], the above term can be simplified as follows:

(A7) $$\begin{array}{cc} \mathrm{Pr}\left\{\gamma_{rp}^{AF}\;<\;R_{p}\right\} & =1-\exp\left(-\varphi_{2}\left(\frac{1}{\lambda_{rp}}\;+\;\frac{1}{\lambda_{br}}\right)\right)\\ \; & \;\times \;\sqrt{\frac{4\varphi_{2}\left(1\;+\;\alpha \varphi_{2}\right)}{\lambda_{br}\lambda_{rp}\alpha }}K_{1}\left(\sqrt{\frac{4\varphi_{2}\left(1+\alpha \varphi_{2}\right)}{\lambda_{br}\lambda_{rp}\alpha }}\right) \end{array}$$

Finally, substituting (A2) and (A5), (A3) and (A7) into (A1), (34) can be obtained.

## Appendix B

**Derivation of (36)**

Equation (36) is derived as follows:

(A8) $$\begin{array}{cc} P_{out}^{AF}\left(S_{B}\right)= & \underset{T_{1}}{\underset{︸}{\left(1-\mathrm{Pr}\{\gamma_{bs1}^{}\geq R_{p},\gamma_{bs2}^{}\geq R_{s}\}\right)}}\\ \; & \times \underset{T_{2}}{\underset{︸}{\left(1-\mathrm{Pr}\{\gamma_{rs1}^{AF}\geq R_{p},\gamma_{rs2}^{AF}\geq R_{s}\}\right)}} \end{array}$$

The first term that is equal to (35) can be simplified as follows:

(A9) $$T_{1}=1-\mathrm{Pr}\{\gamma_{bs1}^{}\geq R_{p},\gamma_{bs2}^{}\geq R_{s}\}=1-\exp\left(-\frac{\theta_{1}}{\lambda_{bs}}\right)$$

As for the second term, by substituting $\gamma_{rs1}^{AF}$ and $\gamma_{rs2}^{AF}$ into it, it can be formulated as follows:

(A10) $$\begin{array}{cc} T_{2}= & 1-\mathrm{Pr}\{\gamma_{rs1}^{AF}\geq R_{p},\gamma_{rs2}^{AF}\geq R_{s}\}\\ \;= & 1\;-\mathrm{Pr}\{\alpha {\left|h_{br}\right|}^{2}\left[\beta {\left|h_{rs}\right|}^{2}a{{}_{s}^{}}^{2}-R_{s}\right]\geq \alpha R_{s}{\left|h_{rs}\right|}^{2}+R_{s},\\ \; & \;\alpha {\;\left|h_{br}\;\right|}^{2}\;\left[\;\beta {\left|h_{rs}\right|}^{2}\left(\;a{{}_{p}^{}}^{2}\;-\;a{{}_{s}^{}}^{2}R_{p}\;\right)\;-\;R_{p}\;\right]\;\geq \;\alpha R_{p}{\left|h_{rs}\right|}^{2}\;\;+\;\;R_{p}\} \end{array}$$

(1) $R_{s}\geq \beta {\left|h_{rs}\right|}^{2}a_{s}^{2},R_{p}<\frac{a_{p}^{2}\beta {\left|h_{rs}\right|}^{2}}{a_{s}^{2}\beta {\left|h_{rs}\right|}^{2}+1}<\frac{a_{p}^{2}}{a_{s}^{2}}$
  (A11)
  $$\begin{array}{cc} T_{2}= & 1-\mathrm{Pr}\left\{{\left|h_{rs}\right|}^{2}>\varphi_{2},{\left|h_{rs}\right|}^{2}\leq \phi_{2},{\left|h_{br}\right|}^{2}\geq \frac{\varphi_{2}\left(1+\alpha {\left|h_{rs}\right|}^{2}\right)}{\alpha ({\left|h_{rs}\right|}^{2}-\varphi_{2})}\right\}\\ = & 1-\int_{\varphi_{2}}^{\phi_{2}}\frac{1}{\lambda_{rs}}\exp\left(-\frac{x}{\lambda_{rs}}\right)\int_{\frac{\varphi_{2}\left(1+\alpha x\right)}{\alpha (x-\varphi_{2})}}^{\infty }\frac{1}{\lambda_{br}}\exp\left(-\frac{y}{\lambda_{br}}\right)dydx\\ = & 1-\int_{\varphi_{2}}^{\phi_{2}}\frac{1}{\lambda_{rs}}\exp\left(-\frac{x}{\lambda_{rs}}-\frac{\varphi_{2}\left(1+\alpha x\right)}{\lambda_{br}\alpha \left(x-\varphi_{2}\right)}\right)dx \end{array}$$
(2) $R_{s}\geq \beta {\left|h_{rs}\right|}^{2}a_{s}^{2},R_{p}\geq \frac{a_{p}^{2}\beta {\left|h_{rs}\right|}^{2}}{a_{s}^{2}\beta {\left|h_{rs}\right|}^{2}+1}$
  (A12)
  $$T_{2}=1$$
(3) $R_{s}<\beta {\left|h_{rs}\right|}^{2}a_{s}^{2},R_{p}<\frac{a_{p}^{2}\beta {\left|h_{rs}\right|}^{2}}{a_{s}^{2}\beta {\left|h_{rs}\right|}^{2}+1}<\frac{a_{p}^{2}}{a_{s}^{2}}$
  (A13)
  $$\begin{array}{cc} T_{2}= & 1-\mathrm{Pr}\{{\left|h_{rs}\right|}^{2}>\varphi_{2},{\left|h_{br}\right|}^{2}\geq \frac{\varphi_{2}\left(1+\alpha {\left|h_{rs}\right|}^{2}\right)}{\alpha ({\left|h_{rs}\right|}^{2}-\varphi_{2})},\\ \; & {\left|h_{rs}\right|}^{2}>\phi_{2},{\left|h_{br}\right|}^{2}\geq \frac{\phi_{2}\left(1+\alpha {\left|h_{rs}\right|}^{2}\right)}{\alpha ({\left|h_{rs}\right|}^{2}-\phi_{2})}\}\\ = & 1-\mathrm{Pr}\left\{{\left|h_{rs}\right|}^{2}>\theta_{2},{\left|h_{br}\right|}^{2}\geq \frac{\theta_{2}\left(1+\alpha {\left|h_{rs}\right|}^{2}\right)}{\alpha ({\left|h_{rs}\right|}^{2}-\theta_{2})}\right\}\\ = & 1-\int_{\theta_{2}}^{\infty }\frac{1}{\lambda_{rs}}\exp\left(-\frac{x}{\lambda_{rs}}\right)\int_{\frac{\theta_{2}\left(1+\alpha x\right)}{\alpha (x-\theta_{2})}}^{\infty }\frac{1}{\lambda_{br}}\exp\left(-\frac{y}{\lambda_{br}}\right)dydx\\ = & 1-\int_{\theta_{2}}^{\infty }\frac{1}{\lambda_{rs}}\exp\left(-\frac{x}{\lambda_{rs}}-\frac{\theta_{2}\left(1+\alpha x\right)}{\lambda_{br}\alpha \left(x-\theta_{2}\right)}\right)dx\\ = & 1-\exp\left(-\theta_{2}\left(\frac{1}{\lambda_{rs}}+\frac{1}{\lambda_{br}}\right)\right)\sqrt{\frac{4\theta_{2}\left(1+\alpha \theta_{2}\right)}{\lambda_{br}\lambda_{rs}\alpha }}\\ \; & \times K_{1}\left(\sqrt{\frac{4\theta_{2}\left(1+\alpha \theta_{2}\right)}{\lambda_{br}\lambda_{rs}\alpha }}\right) \end{array}$$
(4) $R_{s}<\beta {\left|h_{rs}\right|}^{2}a_{s}^{2},R_{p}\geq \frac{a_{p}^{2}\beta {\left|h_{rs}\right|}^{2}}{a_{s}^{2}\beta {\left|h_{rs}\right|}^{2}+1}$
  - If $R_{p}\geq \frac{a_{p}^{2}}{a_{s}^{2}}$,
    (A14) $$T_{2}=1$$
  - If $R_{p}<\frac{a_{p}^{2}}{a_{s}^{2}}$, the following holds:
    (A15) $$\begin{array}{cc} T_{2}= & 1-\mathrm{Pr}\left\{{\left|h_{rs}\right|}^{2}\leq \varphi_{2},{\left|h_{rs}\right|}^{2}>\phi_{2},{\left|h_{br}\right|}^{2}\geq \frac{\varphi_{2}\left(1+\alpha {\left|h_{rs}\right|}^{2}\right)}{\alpha ({\left|h_{rs}\right|}^{2}-\varphi_{2})}\right\}\\ = & 1-\int_{\phi_{2}}^{\varphi_{2}}\frac{1}{\lambda_{rs}}\exp\left(-\frac{x}{\lambda_{rs}}\right)\int_{\frac{\varphi_{2}\left(1+\alpha x\right)}{\alpha (x-\varphi_{2})}}^{\infty }\frac{1}{\lambda_{br}}\exp\left(-\frac{y}{\lambda_{br}}\right)dydx\\ = & 1-\int_{\phi_{2}}^{\varphi_{2}}\frac{1}{\lambda_{rs}}\exp\left(-\frac{x}{\lambda_{rs}}-\frac{\varphi_{2}\left(1+\alpha x\right)}{\lambda_{br}\alpha \left(x-\varphi_{2}\right)}\right)dx \end{array}$$
    where $\phi_{2}=\frac{R_{s}\delta^{2}}{P_{RS}{a_{s}}^{2}}=\frac{R_{s}}{\beta {a_{s}}^{2}}$ and $\theta_{2}=\max\left(\varphi_{2},\phi_{2}\right)$.

To sum up, the outage probability can be derived as (36).
